# Successful thromboaspiration of bilateral pulmonary thromboembolism in a Fontan circulation patient: a case report

**DOI:** 10.1093/ehjcr/ytae190

**Published:** 2024-06-24

**Authors:** Èlia Rifé-Pardo, Pau Rello, Antonia Sambola, Blanca Gordon

**Affiliations:** Department of Cardiology, Research Institute Vall d’Hebron University Hospital, CIBER-CV, Passeig Vall d'Hebrón 119-129, 08035 Barcelona, Spain; Department of Cardiology, Research Institute Vall d’Hebron University Hospital, CIBER-CV, Passeig Vall d'Hebrón 119-129, 08035 Barcelona, Spain; Department of Cardiology, Research Institute Vall d’Hebron University Hospital, CIBER-CV, Passeig Vall d'Hebrón 119-129, 08035 Barcelona, Spain; Department of Cardiology, Research Institute Vall d’Hebron University Hospital, CIBER-CV, Passeig Vall d'Hebrón 119-129, 08035 Barcelona, Spain

**Keywords:** Case report, Pulmonary thromboembolism, Fontan, ACHD, Percutaneous thrombectomy

## Abstract

**Background:**

Fontan surgery aims to palliate univentricular congenital heart diseases in which biventricular repair is not feasible. A large spectrum of early and late complications has been described in literature. However, pulmonary thromboembolism represents a rare complication in these patients, leading to a scarcity of evidence regarding diagnosis and treatment strategies.

**Case summary:**

We present a case of a 27-year-old woman born with a complex cyanotic congenital heart disease, namely pulmonary and tricuspid stenosis with subaortic interventricular communication and atrial septal defect, who underwent palliation surgery with Blalock–Taussig shunt, bidirectional Glenn, and extracardiac Fontan. She developed acute respiratory failure and was admitted to the hospital, being diagnosed with bilateral thromboembolism. Since she was haemodynamically stable, initially, a conservative approach was chosen. However, due to no clinical improvement, she subsequently underwent bilateral thromboaspiration with restoration of pulmonary circulation.

**Discussion:**

Due to the unique Fontan pathophysiology, the possible physiological and clinical implications of pulmonary thromboembolism in this condition are profound. Thus, care and imaging tests in specialized centres are important as the management of these patients is different from those with biventricular physiology.

**Learning points**

- Pulmonary thromboembolism is a rare but potentially fatal complication in Fontan circulation patients.
- Rescue percutaneous thrombectomy may be considered in selected cases.

## Introduction

Fontan surgery was originally conceived as a repair of univentricular hearts in which a biventricular correction cannot be attained. Caval veins are directly connected to the pulmonary arteries separating completely the systemic and pulmonary systems. Therefore, any resistance variation of the Fontan circuit can have significant haemodynamic consequences.^1^

Since first performed in 1968, several technique modifications have been introduced leading to a significant improvement in outcomes and life expectancy.^2–4^ However, multiple complications continue to be encountered in early and late follow-up, highlighting multifocal thrombosis as one of them. Numerous elements contribute to this state of hypercoagulability: blood stasis secondary to low flow velocity, anomalies in procoagulant and anticoagulant factors, and increase in platelet reactivity or hepatic dysfunction, among others.^2^

We present a case of bilateral pulmonary thromboembolism (PTE) in a patient with Fontan circulation.

## Summary figure

**Table:** 

Timeline	Events
Initial presentation	Sudden dyspnoea and chest pain.
Initial paraclinic investigation	Respiratory failure (RF), haemodynamic stability, and evidence of bilateral PTE and Fontan thrombosis in another centre chest computed tomography (CT).
Day 0	Evidence of bilateral PTE but with permeable Glenn and Fontan when repeated chest CT using a Fontan imaging protocol. Initiation of unfractionated heparin (UFH). Lower limb Doppler ruled out deep vein thrombosis.
Day +3	Persistent RF. Evidence of pulmonary infarcts in new CT. Percutaneous thromboaspiration.
Day +11	Improvement in arterial repletion defects and pulmonary opacities in a control CT.
Day +13	Discharged with low-molecular-weight heparin (LMWH) treatment.
After discharge	Remained asymptomatic.

## Case presentation

We present a 27-year-old Caucasian woman admitted to a district general hospital with acute dyspnoea and chest pain. She was prenatally diagnosed with a complex cyanotic congenital heart disease, namely a severe pulmonary and tricuspid valve stenosis with a hypoplastic right ventricle with a subaortic ventricular septal defect and a broad atrial septal defect. It is therefore a univentricular heart with a dominant left ventricle morphology. A few weeks after birth, she underwent modified Blalock–Taussig shunt palliation, and at 5 years of age, she underwent a bidirectional Glenn, pulmonary branch enlargement, pulmonary artery banding, and Blalock–Taussig shunt closure. At 12 years old, she underwent an extracardiac Fontan with a non-fenestrated conduit and complete closure of the main pulmonary artery. During follow-up, both diastolic and systolic functions of the systemic ventricle were preserved, and significant valvulopathies were not identified. Furthermore, Fontan pressures were normal in the latest catheterism done at 24 years old. As complications, she developed non-cirrhotic congestive hepatopathy and lower extremity venous insufficiency. In addition to her cardiologic background, she was under review for a mild pancytopenia and anaemia secondary to hypermenorrhoea. Her regular medications were aspirin, omeprazole, sertraline, alprazolam, and oral desogestrel. Her ambulatory oxygen saturations were around 96–98% at ambient air. One month before hospitalization, she had been scratched by a cat at the right lower extremity. She was eventually diagnosed with cellulitis caused by an unidentified microorganism, receiving a short treatment of amoxicillin–clavulanic acid. Our unit was not notified of this event until admission.

She presented to the emergency department due to acute dyspnoea and chest pain. On examination, she was haemodynamically stable, heart rate of 60 b.p.m., and apyrexial. She was taquipneic with an oxygen saturation around 95% requiring a fraction of inspired oxygen of 100%, with no other relevant findings at physical examination.

The electrocardiogram showed a sinus rhythm with non-specific diffuse negative T-waves, already described in previous records. An urgent chest CT showed evidence of bilateral PTE and Fontan thrombosis. Therefore, the patient was transferred to our centre, being the referral hospital for congenital heart disease.

**Figure 1:**
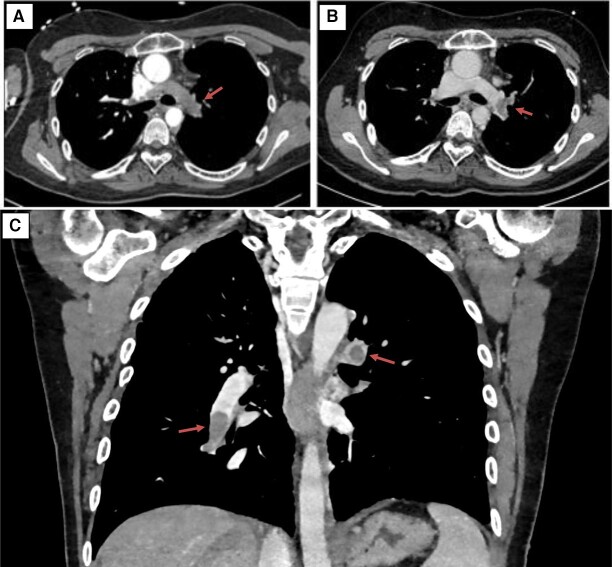
(*A* and *B*) Comparison between chest computed tomography performed without (left) and with (right) Fontan protocol. In both images, pulmonary thromboembolism is present (arrows). (*C*) A coronal view of the bilateral pulmonary thromboembolism (arrows).

**Figure 2:**
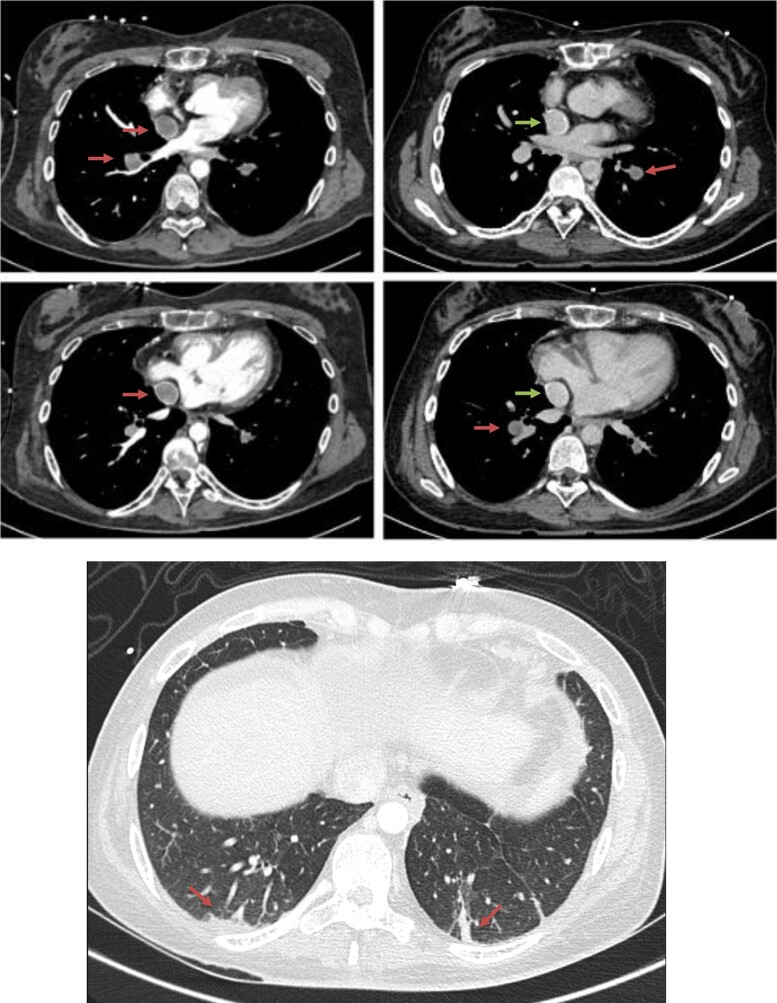
Comparison between chest computed tomography performed without (left) and with (right) Fontan protocol. Left images show pulmonary thromboembolism and Fontan thrombosis (arrows) while right images, taken with a very delayed acquisition, evidence permeability of the Fontan circuit (upper arrows) with persistance of pulmonary thromboembolism (lower arrows). The lower image (lung window) shows bilateral pulmonary infarcts (arrows).

On her arrival, a new chest CT was taken using our centre Fontan imaging protocol. Firstly, a bolus test using 10 mL of contrast is used in order to define the venous and arterial phases. Secondly, a high-pitch helical venous acquisition and a significantly delayed (120 s) arterial acquisition are taken. Using this protocol, bilateral PTE was documented but with full permeability of the Glenn and Fontan circuits (*Figures 1* and *2*). The echocardiography found no changes compared with previous ones maintaining both Fontan and Glenn flow laminar with low velocities and respiratory variations.

She was admitted to the intensive coronary unit, and, due to haemodynamic stability, a conservative approach was pursued and UFH treatment was initiated. We aimed for an activated partial thromboplastin time of 60–85 s. The initial laboratory workup revealed normal levels of haemoglobin, platelet count, renal and liver function, as well as negative troponin I. Lower limb Doppler ruled out deep vein thrombosis.

**Figure 3:**
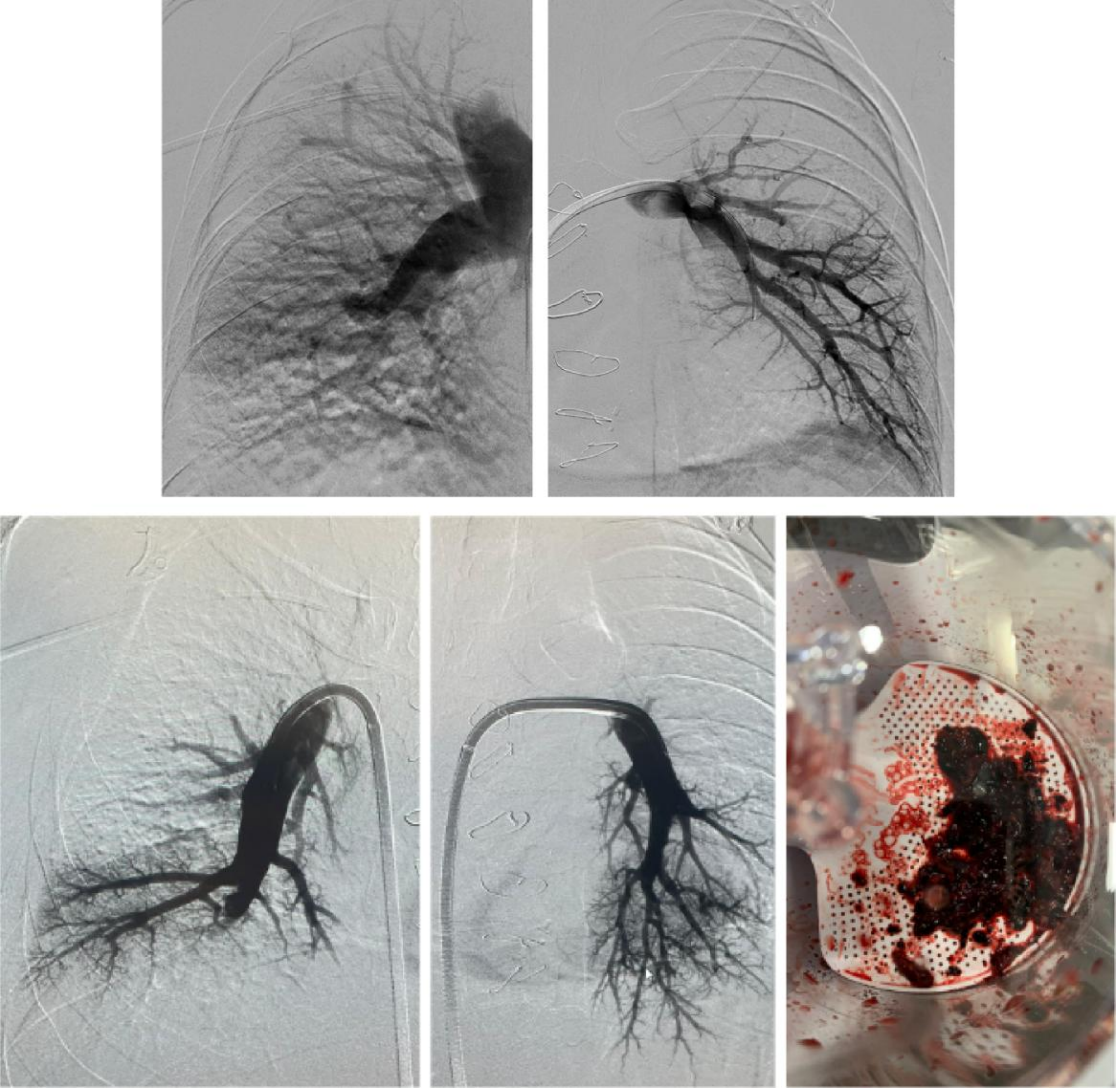
Pre (upper) and post (lower) angiographic images of the percutaneous thromboaspiration. The last image shows the amount of thrombus that was removed.

After 48 h, the patient remained with high oxygen requirements, leading to the performance of a new CT on Day 3, which revealed the development of multiple bilateral pulmonary infarcts. These results made clear that a conservative strategy could no longer be endured. Therefore, a percutaneous thromboaspiration was successfully performed using a right femoral access and a Penumbra Inc. Indigo Lightning 12 system, with the administration of 200 000 IU of urokinase (*Figure 3*).

**Figure 4:**
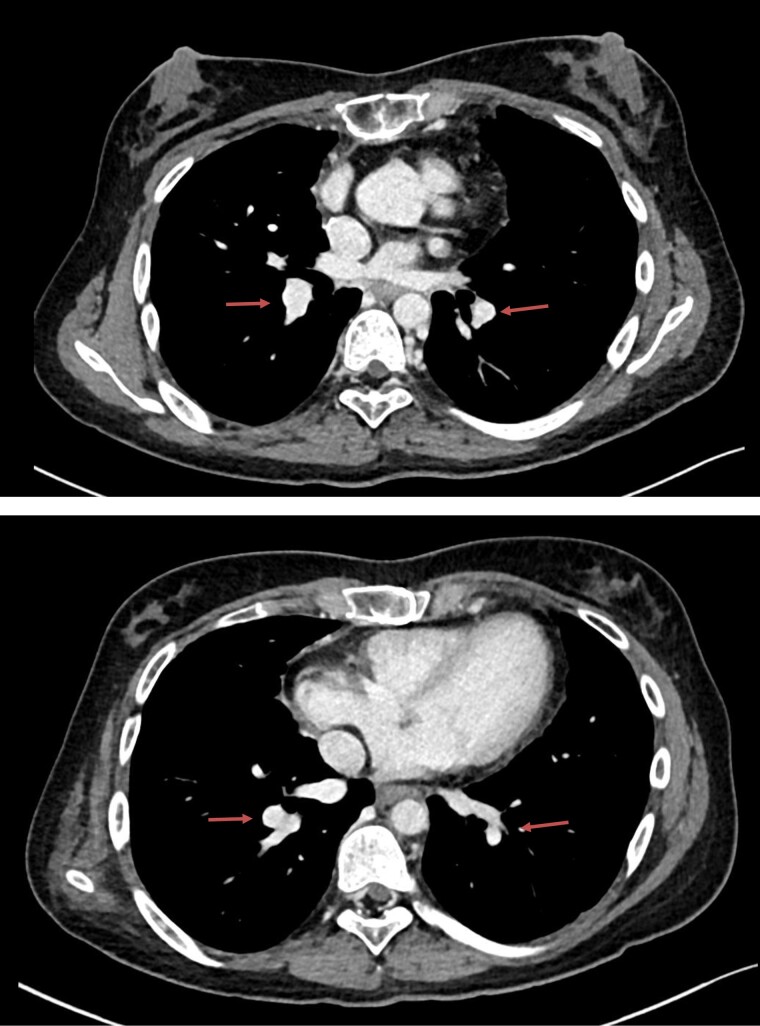
Post percutaneous thromboaspiration control chest computed tomography with resolution of pulmonary thromboembolism (arrows).

Oxygen supplementation was withdrawn within a few days, and UFH was switched to weight-adjusted LMWH treatment. One week after the thromboaspiration, a new chest CT was performed documenting the resolution of PTE and improvement on pulmonary opacities (*Figure 4*). Moreover, significant systemic–pulmonary veno-venous collateral vessels were seen, which probably helped maintain cardiac output and haemodynamic stability during admission.

She was discharged after 13 days of hospitalization on chronic anticoagulation initially with LMWH, switching to vitamin K antagonists (VKAs) after 6 months. Aspirin and desogestrel were withdrawn. The patient has remained asymptomatic since this event and has not presented new thrombotic complications.

## Discussion

We present a very rare case with challenging diagnostic and treatment decisions. Fontan surgery was originally conceived as a palliative procedure for univentricular hearts in which a biventricular correction was not feasible. The surgical technique improvements and the better follow-up over the years have led to an important improvement in survival.^3,4^ Due to the anatomical modifications caused by these interventions, any resistance variation of the Fontan circuit can have significant consequences. Therefore, the presence of thrombus at the arterial pulmonary system can have a huge repercussion in these patients.^1^ Moreover, most of the factors associated with worse prognosis in patients with conventional anatomy have not been validated in patients with Fontan circulation.^5^

Thrombosis is one of the many major complications that can be encountered in these patients and can considerably impact morbidity and mortality.^1,2,6^ Many factors can contribute to an increase in thrombogenicity. Regarding primary preventive treatment, patients with a clear indication for anticoagulation (for example previous thrombotic history or atrial arrhythmia) should receive chronic oral anticoagulation.^6,7^ In all other cases, there is controversy in the best antithrombotic strategy. Since our patient had not been diagnosed with any of these risk factors, was being studied for mild pancytopenia, and had hypermenorrhoea, she was only on a low-dose aspirin regimen. Furthermore, after hospital discharge, VKAs were chosen due to the characteristics of the patient as the international normalized ratio can be periodically assessed.

Diagnosis of PTE in these patients is challenging, requiring a very late acquisition due to the very slow flow in the circuit.^8,9^ Also, little is known about the management, and, in this scenario, the decision of the best individualized treatment is difficult. In our case, surprisingly, there was haemodynamic stability, which we believe was due to the significant venous–venous circulation that had developed between the systemic venous circuit and the pulmonary veins. This allowed ventricular preload to be maintained at the cost of greater desaturation. For this reason, we opted for initial conservative management, allowing time to see the response to anticoagulant treatment. Eventually, due to lack of clinical improvement, a percutaneous thromboaspiration was successfully performed allowing oxygen weaning. This technique has been performed successfully in Fontan circulation in previous cases when medical treatment was not successful,^10,11^ being less aggressive when compared to surgical embolectomy. Our case, along with the previous literature, emphasizes that this should be an option to consider in these situations, taking into account the numerous previous surgeries and sternotomies.

## Data Availability

No new data were generated or analysed in support of this research.

## Lead author biography

Èlia Rifé received her medical degree from the University of Barcelona in 2020. Currently, she is in her third year of residency in cardiology at Hospital Vall d'Hebron in Barcelona.

**Consent:** The authors confirm that written consent for submission and publication of this case report, including images and associated text, has been obtained from the patient in line with COPE guidance.

**Funding:** None declared.
